# Sustainable Alkali Activation: The Role of Water- and Alkali-Treated Sisal Leaf Wastewaters in Solid- Waste-Based Composite Synthesis

**DOI:** 10.3390/ma17153838

**Published:** 2024-08-02

**Authors:** Liang Li, Hongqi Yang, Xianhui Zhao, Haoyu Wang, Renlong Zhao

**Affiliations:** 1 School of Civil Engineering, Tianjin Renai College, Tianjin 301636, China; 223035@tjrac.edu.cn; 2 CCCC First Harbor Consultants Co., Ltd., Tianjin 300220, China; hongqiyang0707@126.com; 3 School of Civil Engineering, Hebei University of Engineering, Handan 056038, China; zhaoxianhui@hebeu.edu.cn; 4 Zhongtu Dadi International Architectural Design Co., Ltd., Shijiazhuang 050000, China; hbdd86990816@126.com

**Keywords:** alkali-activated composites, sisal, wastewater, slag, fly ash, mechanical strength, microstructure

## Abstract

The intricate composition of wastewater impedes the recycling of agricultural and industrial effluents. This study aims to investigate the potential of sisal leaf wastewater (SLW), both water-treated (WTSLW) and alkali-treated (ATSLW), as a substitute for the alkali activator (NaOH solution) in the production of slag-powder- and fly-ash-based composites, with a focus on the effects of WTSLW substitution ratios and sisal leaf soaking durations. Initially, the fresh properties were assessed including electrical conductivity and fluidity. A further analysis was conducted on the influence of both WTSLW and ATSLW on drying shrinkage, density, and mechanical strength, including flexural and compressive measures. Microstructural features were characterized using SEM and CT imaging, while XRD patterns and FTIR spectra were employed to dissect the influence of WTSLW substitution on the composite’s products. The results show that incorporating 14 wt% WTSLW into the composite enhances 90-day flexural and compressive strengths by 34.8% and 13.2%, respectively, while WTSLW curtails drying shrinkage. Conversely, ATSLW increases porosity and decreases density. Organic constituents in both WTSLW and ATSLW encapsulated in the alkaline matrix fail to modify the composites’ chemical composition. These outcomes underscore the potential for sustainable construction materials through the integrated recycling of plant wastewater and solid by-products.

## 1. Introduction

Wastewater poses a significant global challenge as it becomes contaminated with various pollutants, rendering it unsuitable for its intended use [1]. Industrial, agricultural, and domestic activities are the primary sources of wastewater, carrying substantial health risks due to the presence of harmful microorganisms [2]. For instance, agricultural wastewater is generated when plant stalks, stems, roots, and leaves undergo fermentation after prolonged soaking in rainwater and river water, resulting in an acidic waste liquid that degrades water quality [3,4]. On the other hand, industrial wastewater presents challenges in terms of purification and disposal due to its complex contaminants [5,6]. The discharge of industrial and domestic wastewater from urban areas globally contaminates extensive water areas, surpassing the size of the water bodies themselves [7]. Consequently, overall, the sustainable management and disposal of wastewater is essential for addressing water scarcity, protecting public health, and preserving environmental quality.

In the field of materials development, various natural vegetation and plant fibers, including cotton straw [8], barley straw [9], wheat straw [10], corn stem [11], sisal fiber [12,13], etc., have undergone chemical treatments before incorporation into composite materials [14]. Among these treatments, including chemical treatments, are alkali treatment, acetylation, silane treatment, and benzoylation treatments [15,16]; alkali treatment has gained popularity due to its simplicity and effectiveness. It involves the use of alkaline solutions, such as NaOH and KOH, to eliminate non-cellulosic components like hemicellulose, pectin, polysaccharides, lignin, and carbohydrates from natural fibers, resulting in improved hydrophobic properties and the conversion of hydrophilic characteristics to hydrophobic ones [17,18].

While previous studies [8,14,19] have primarily focused on the alkali treatment of natural plant fibers, straw, stem, and sawdust, as well as the preparation and characterization of composite materials, the attention given to alkaline wastewater generated from the alkali treatment process has been insufficient, and its technical recycling has been neglected. Alkali-treated plant wastewaters contain complex constituents, including inorganic pollutants, plant nutrients, carbohydrates, and lignin. These contaminants contribute to increased osmotic pressure, organic compounds, decreased dissolved oxygen, and harm to plants, fish, and other animals. Furthermore, the breakdown of organic molecules in alkali-treated plant wastewater leads to the production of odorous compounds such as hydrogen sulfide, ammonia, and mercaptans. Given the high liquor ratio involved in the alkali treatment process [20] and subsequent washing with distilled water [21], a significant volume of alkali-treated plant wastewater is generated, necessitating the development of an efficient method for their reuse.

Alkali-activated materials offer a unique solution as inorganic cementitious materials with a 3D polymeric structure [19,22]. These materials are synthesized by combining industrial silica–alumina wastes (e.g., fly ash, red mud, and slag) with highly alkaline activators [23,24]. Utilizing diverse solid wastes in the production of alkali-activated materials requires careful consideration of their chemical components as they significantly influence the physical, mechanical, and microstructural properties, as well as the durability of the materials [25,26]. Alkali-activated materials exhibit favorable characteristics compared to conventional Portland cement, including high early strength [27], surface hardness [28], chemical corrosion resistance [29], as well as sequestration of harmful ions [30]. In a previous study, it was observed that sisal leaves had a more pronounced impact on modifying the properties of composite materials compared to recycled wastewater from corn stems, poplar leaves, polar stems, sisal leaves, and corn stems [3]. Moreover, it is noteworthy that wastewaters derived from water-treated natural plants are acidic [4,31], while those derived from alkali-treated natural plants remain highly alkaline [18]. The chemical composition of natural plant fibers mainly consists of cellulose and lignin [32]. After alkali treatment, the resulting alkaline solutions contain organic substances classified as chemical wastewater. However, the effects of water-treated and alkali-treated plant wastewaters are still unclear in regard to the synthesis and properties of alkali-activated materials based on diverse solid wastes.

The objective of this study is to investigate the effect mechanism of water-treated sisal leaf wastewater (WTSLW) and alkali-treated sisal leaf wastewater (ATSLW) in substituting alkali activators (NaOH solution). Initially, this study explores the effects of WTSLW and ATSLW on the fresh and hardened properties of alkali-activated solid-waste-based composites, encompassing fluidity, electrical conductivity, density, drying shrinkage, flexural, and compressive strengths. Subsequently, this research including SEM, EDS, and CT tests delves into the influence of WTSLW and ATSLW on the microstructure of alkali-activated solid-waste-based composites. Furthermore, the mineral composition and products of composites incorporating WTSLW were detected through XRD and FTIR tests, thereby focusing on revealing the roles and advantages of WTSLW and ATSLW. The main novelty is that two types of wastewater, WTSLW and ATSLW, are introduced into alkali-activated solid-waste-based materials. The findings will provide valuable insights into the technical dual application of sisal leaf wastewater and solid waste in alkali-activated composites, and they will contribute to the standardization process of plant wastewater recycling.

## 2. Materials and Methods

### 2.1. Raw Materials

The experimental study employed a set of raw materials, encompassing binder material, fine aggregate, sisal leaf wastewater, and an alkali activator. Specifically, slag powder (SP), red mud (RM), and fly ash (FA) were carefully selected as silica–aluminum binder materials. The use of standard sand as the fine aggregate was consistent throughout this study. Two distinct types of sisal leaf wastewater were obtained: one derived from water treatment and the other resulting from alkali treatment of mature sisal leaves. The alkali activator primarily consisted of a NaOH solution, with the additional utilization of alkali-treated sisal leaf wastewater as an alternate alkali activator.

#### 2.1.1. Binder Materials

The present experiment utilized #S95 grade slag powder (SP) sourced from Gongyi City (Gongyi, China). The SP had a fineness of 800 mesh and a specific gravity of 1299 m^2^/kg. The pH value of the SP was measured at 11.45, while its electrical conductivity stood at 0.44 mS/cm. The pH and electrical conductivity measurements were obtained using a solid-water mass ratio of 1:5. Red mud (RM), also obtained from Gongyi City (Gongyi, China), was utilized. The RM had a fineness of 500 mesh. Fly ash (FA) was sourced from a power plant located in the Handan Economic and Technological Development Zone in Handan City (Handan, China). The fineness of FA met the Class II fly ash standard, with a #325-mesh square hole sieve margin of 22%. The specific gravity of FA was measured at 490 m^2^/kg, while the pH value and electrical conductivity were 8.30 and 2.75 mS/cm, respectively.

**Figure 1:**
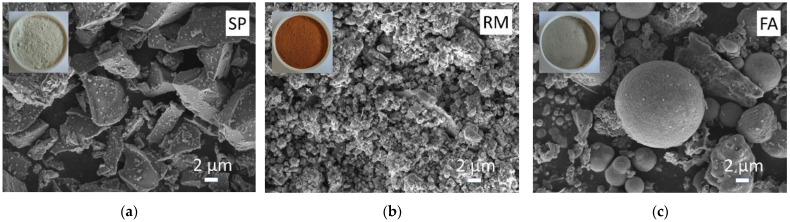
SEM images of binder materials: (**a**) slag powder (SP), (**b**) red mud (RM), (**c**) fly ash (FA).

Figure 1 presents scanning electron microscope (SEM) images of SP, RM, and FA, while Table 1 provides their respective chemical compositions. SP exhibits irregular granular and lamellar structures with fine particles, a loose structure, and high hydration activity [33,34]. Its chemical composition primarily consists of SiO_2_, Al_2_O_3_, CaO, and MgO. Similarly, RM displays irregular granular structure, fine particles, loose structure, and high alkalinity, dominated by SiO_2_, Al_2_O_3_, and Fe_2_O_3_. FA exhibits spherical glass beads and irregular granular structure, characterized by low activity and mainly comprising SiO_2_ and Al_2_O_3_. The FA belongs to the low-calcium ash category with a CaO content of 5.40%. SP, RM, and FA share common components, including SiO_2_ and Al_2_O_3_, which serve as raw materials for alkali activation and are crucial for the occurrence of alkali activation and geopolymer formation.

Alkali-activated FA geopolymer typically exhibits a 28-day compressive strength of less than 10.0 MPa at room temperature [35,36]. However, incorporating SP and RM can enhance the mechanical properties of FA-based geopolymers [37,38]. Consequently, this study aimed to synthesize multi-solid-waste materials through alkali activation using SP, RM, and FA complexes, to achieve improved curing effects at low alkaline concentrations.

#### 2.1.2. Fine Aggregate

ISO standard sand (Produced by Xiamen Aisiou Company, Xiamen, China) was used as fine aggregate, and the index meets the requirements according to the standard GB/T 17671-2021 [39].

#### 2.1.3. Sisal Leaf Wastewater

**Figure 2:**
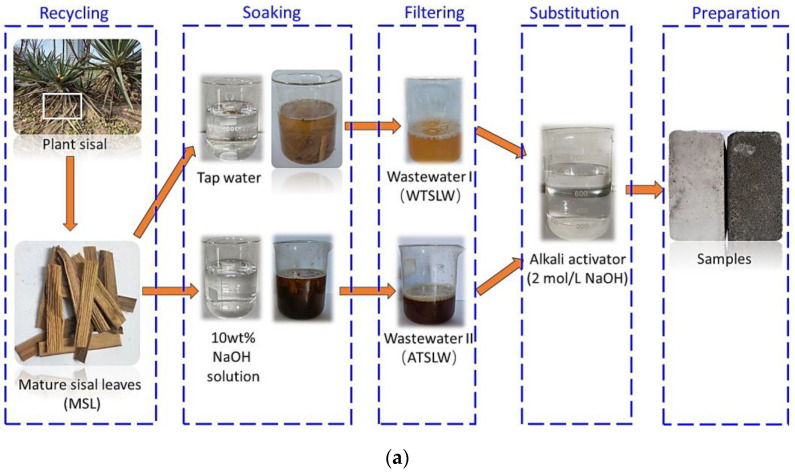

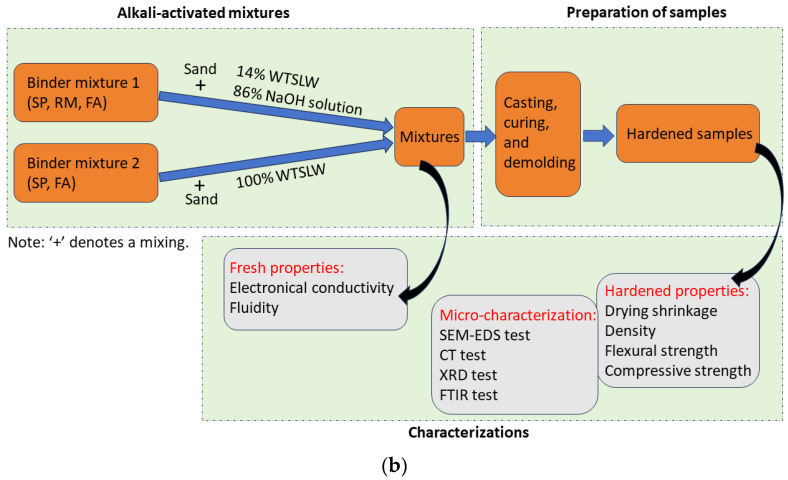
(**a**) Recycling and treating of two types of sisal leaf wastewater with treatment by tap water and NaOH solution as substitution of alkali activator for sample preparation. WTSLW denotes water-treated sisal leaf wastewater, and ATSLW denotes alkali-treated sisal leaf wastewater. (**b**) Flowchart of the experimental characterization methods.

Sisal leaf wastewater was selected for use, which has a more pronounced impact on modifying the properties of composites [3]. Two methods were employed to obtain sisal leaf wastewater, as illustrated in Figure 2.

Water treatment: Mature sisal leaves sourced from Shijiazhuang, Hebei Province, were utilized for this process. The sisal leaves were cut into 5–6 cm lengths and placed in a glass container for soaking. Water sourced from the Handan Economic and Technological Development Zone, Handan, China, with a pH of 7.20 ± 0.05 and an electrical conductivity (EC) of 25.00 μs/cm was used. The sisal leaves were soaked in glass containers with tap water for 5 days, with a liquor ratio of 1:20. Subsequently, the mixture was filtered to obtain water-treated sisal leaf wastewater (labeled WTSLW). The WTSLW had a measured pH of 5.87 and an EC of 2.78 mS/cm, indicating its acidic nature.

Alkali treatment: The use of an alkali solution enhances the rapid extraction of impurities from sisal leaves compared to water. Analytically pure NaOH pellets (purity > 98%) were mixed with tap water to prepare a 10 wt% NaOH solution for the treatment process [40]. Additionally, mature sisal leaves were immersed in the 10 wt% NaOH solution for various durations (1 ± 0.2 h, 5 ± 0.2 h, and 8 ± 0.2 h), followed by filtration to obtain alkali-treated sisal leaf wastewater (labeled ATSLW). Due to the presence of NaOH, the ATSLW exhibited strong alkalinity (pH > 13.98). Furthermore, a comparison was made with NaOH solution that did not undergo immersion treatment.

#### 2.1.4. Alkali Solution

A 2.0 mol/L NaOH solution was prepared by dissolving a commercially available NaOH pellet into tap water. The NaOH pellet, obtained from Tianjin Aopusheng Chemical Co., Ltd., Tianjin, China, had an analytical purity higher than 98%. It was found effective for pretreating sisal leaves to enhance surface bonding within the designated soaking duration [41,42,43]. Furthermore, considering the high alkalinity of the ATSLW, it was also considered an alkaline activator.

### 2.2. Preparation of Alkali-Activated Samples

#### 2.2.1. Effect of Wastewater Obtained from Water-Treated Sisal Leaves

The addition ratios of WTSLW and the mixing ratios of the alkali-activated composites are shown in Table 2. The mortar samples SRF1, SRF2, and SRF3 were used to determine the physical and mechanical properties. Paste samples PSRF1 and PSRF3 were used to carry out microscopic tests. The paste samples take slightly lower values for the liquid-to-solid ratio as no fine aggregate is added. The samples DSRF1 and DSRF3 were used to determine the drying shrinkage.

The three binders, SP, RM, and FA, were first mixed well to form a solid mixture. Standard sand was then added to it. Once well mixed, a NaOH solution was added to the stirrer in successive stages and stirred slowly for three minutes. A design proportion of WTSLW was then added to the system. To keep the liquid-to-solid ratio constant, an additional NaOH solution was added as a supplement. Then, it was stirred slowly for two minutes to obtain a fresh alkali-activated mixture. This mixing method is slightly different from the standard preparation of mortar [39]: firstly, the standard sand is added much earlier, secondly, the sand is mixed for a longer period. The former solves the problem of the difficulty in controlling the flowability after the WTSLW is added due to the water absorption of the fine aggregate, while the latter aims to facilitate better integration of the sisal leaf wastewater into the binders and to obtain a more homogeneous mixture. The fresh alkali-activated mixture was then poured into different-sized molds to allow for different experimental studies. The specimens were sealed and placed at room temperature (above 50% humidity) for 6 h and then demolded. Curing was then continued in the same way until the required age.

#### 2.2.2. Effect of Wastewater Obtained from Alkali-Treated Sisal Leaves

To explore the effect of ATSLW as an alkali activator, a highly reactive mixture containing SP and FA was selected to use as a binder material. The combined use of slag and fly ash yields a stronger alkali-activated sample than fly ash alone [38,44]. In addition, the soaking duration of sisal leaves under alkaline solutions influences the composition of the obtained ATSLW [42,43,45]. To investigate the effect of different treatment durations of alkali solutions on the properties of the synthesized composites, four groups of samples were designed for this work (Table 2). Among them, sample SF1 was used as a control, which was prepared by using untreated NaOH solution for the preparation of alkali-activated composite samples. The mature sisal leaves were soaked, respectively, in a 10 wt% NaOH solution for various durations in a liquor ratio of 1:20, and the ATSLW was obtained by filtration. Then, the ATSLW was directly used as an alkali activator to prepare samples, where samples SF2, SF3, and SF4 were prepared by using the ATSLW with three different treatment durations of 1 ± 0.2 h, 5 ± 0.2 h, and 8 ± 0.2 h, respectively. Mortar samples DSF1, DSF2, DSF3, and DSF4 were manufactured in a binder–sand ratio of 1:2.0 and used to determine the drying shrinkage.

According to GB/T 17671-2021 (ISO) [39], the SP, FA, and standard sand were first mixed well into a solid mixture. Then, the ATSLW was added to the solid mixture at a suitable liquid-to-solid ratio, and it was stirred slowly for five minutes. The fresh mixture was poured into the mold and allowed to rest at room temperature (above 50% humidity) and then demolded after a curing age of seven days under the sealed condition. Demolding after seven days is performed to prevent disturbance to the early structure of the sample. After demolding, all samples were continuously conserved under identical conditions until the required measurement age.

In the above preparation methods (Table 2), the vibration step of the fresh sample was not performed. This is because vibration can cause the alkaline solution and wastewater to uplift and even cause segregation, which impacts the homogeneity of the sample and causes greater deviations in the results of the samples. In addition, the absence of vibration made it more convenient to observe and characterize the microstructure and pore characteristics of the composites doped with sisal wastewater.

### 2.3. Experimental Program

#### 2.3.1. Characterization of Fresh Properties

The physical and chemical properties of the fresh mixture were characterized by assessing the electrical conductivity (EC) and fluidity. The EC was ascertained through a DDS-307A meter, with a measurement spectrum extending to 100 mS/cm precision in 0.01 mS/cm increments. Standard 10 cm^−1^ probes facilitated the EC assessments. To capture sufficient variability, these measurements were performed at six strategically distributed points within the mixing vessel, with the probes set at a consistent 1 to 2 cm submersion depth. The calculated average EC represented the overall EC of the mixture. Subsequently, the mixture was transferred to a truncated conical vessel to determine its flow diameter through the GB/T 2419-2005 [46] jump table methodology. The averaged flow diameter figures were derived from triplicate samples. They were executed immediately post-mixing before the material was poured into molds.

#### 2.3.2. Assessment of Hardened Properties

(1) Determination of drying shrinkage and density

Samples, each measuring 25 mm × 25 mm × 280 mm, had their shrinkage meticulously recorded over 28- and 90-day intervals using the BC156-300 ratio–length meter (from Lisheng, Cangzhou, China), adhering to the Chinese standard JC/T 603-2004 [47]. For reliability, six duplicates were measured under identical experimental setups. Concurrently, the density of the samples was evaluated at the respective time points through the mass-to-volume method [36], with the resultant values averaged from six parallel samples measured under uniform conditions.

(2) Evaluation of flexural and compressive strengths

The evaluation of flexural and compressive strengths provided insight into the age effect and stability of the alkali-activated samples. Samples, each with dimensions of 40 mm by 40 mm by 160 mm, were tested at 28 and 90 days to examine the progression of their mechanical properties. The flexural strength was determined by averaging the measures from three parallel samples. This metric was juxtaposed against the compressive strength, which was synthesized from the average of six parallel samples. The testing utilized a servo-controlled mechanical testing system (model YAW-S from Sansi Zongheng, Shenzhen, China) with a peak capacity of 300 kN. The compression tests were executed at a rate of 2400 N/s, contrasting the flexural tests conducted at 50 N/s, according to the standard GB/T 17671-2021 [39].

#### 2.3.3. Analysis of Microstructural Features

(1) Surface morphology and microstructure examination

To further characterize the 90-day samples and binder materials, scanning electron microscopy (SEM) coupled with energy-dispersive spectroscopy (EDS) tests were conducted to scrutinize the morphology and microstructure of samples (PSRF1, PSRF3, SF1, and SF4). The SEM equipment (TESCAN MIRA LMS model) was produced in the Czech Republic and the EDS equipment (Smartdex model) was produced in Oxford, England. To enhance conductivity for the SEM analysis, the specimens were coated with a thin layer of gold. The elemental composition of select areas within the samples was elucidated by using EDS spectra. In addition, X-ray computed tomography (CT) was utilized to quantify the porosity of the mortar specimens. A 40 mm × 40 mm × 40 mm cubic sample was scanned at 220 kV and 200 A after the flexural strength tests. The resulting 0.1 mm resolution projections facilitated a three-dimensional (3D) analysis of pore morphology, accomplished by using the VG Studio Max v3.2 software [48].

(2) Identification of mineral phases and products

To further identify the effect of WTSLW on the solid-waste-based composites, the samples PSRF1 and PSRF3 were further researched. An X-ray diffraction (XRD) analysis was performed by using a Rigaku SmartLab-SE model (Tokyo, Japan) with Cu-Kα radiation to discern the mineral phases present within the samples. The scan was executed at a rate of 2°/min across the 2θ range of 10° to 80°. As for the 90-day samples and binder materials, they were subjected to oven drying for 48 h at 45 °C and then finely ground to achieve a particle size of 2.0 μm. The preparation for Fourier-transform infrared spectroscopy (FTIR) involved blending 1.3 ± 0.001 mg of the sample with 130 mg of KBr reagent and applying pressure at 20 MPa. Subsequently, the FTIR spectrometer (Thermo Scientific Nicolet iS20 model, Shanghai in China) was employed to identify characteristic vibration peaks and to analyze the formation products within the wavenumber range of 400 to 4000 cm⁻^1^.

## 3. Results and Discussion

### 3.1. Effects of Sisal Leaf Wastewater on Electrical Conductivity and Fluidity

**Figure 3:**
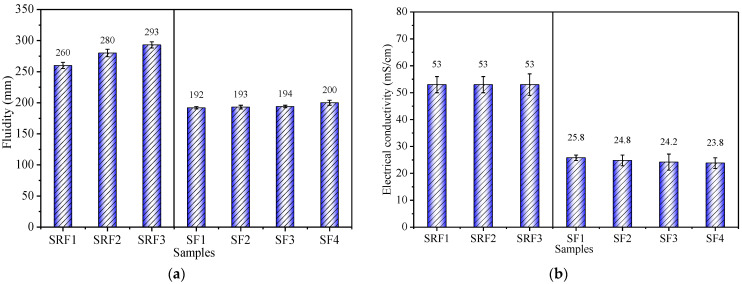
(**a**) Fluidity and (**b**) electrical conductivity of alkali-activated mixtures with sisal leaf wastewater.

This study investigates the effects of sisal leaf wastewater (WTSLW and ATSLW) on the fluidity and electrical conductivity of alkali-activated mixtures (Figure 3). WTSLW replaces the alkali activator from 0% to 14%, and fluidity increases, with SRF3 showing a 12.7% increase compared to SRF1. However, when ASTLW fully replaces the activator, fluidity only slightly increases with soaking duration, with SF4 increasing by 4.2% compared to SF1. This could be due to the dissolved organic impurities from wastewater slowing down the gelation speed of the mixture. Longer soaking durations lead to more dissolved impurities, delaying gelation and hardening.

WTSLW’s substitution for the alkali activator does not significantly alter the electrical conductivity of the fresh mixture. Even when ATSLW fully replaces the activator, conductivity only slightly decreases with increased soaking duration, with SF4 showing a 7.8% decrease compared to SF1. WTSLW forms an acidic solution upon soaking, reducing the alkalinity of the system. However, its low electrical conductivity (2.78 mS/cm) compared to the alkali-activated system (53.0 mS/cm) means that the Na^+^ and OH^-^ ions from the NaOH solution are the primary contributors to conductivity. Thus, 14% WTSLW substitution does not impact conductivity. When ATSLW acts as an activator, conductivity slightly decreases with increased soaking duration due to the dissolution of organic impurities from the sisal leaves [49]. These substances, mainly existing in molecular form [50], slightly reduce the ion concentration responsible for conductivity.

In conclusion, WTSLW and ATSLW substitution for the alkali activator in the synthesis of alkali-activated solid-waste composites maintains the ion concentration level but hinders the early gelation speed to some extent.

### 3.2. Effects of Sisal Leaf Wastewater on Drying Shrinkage and Density

**Figure 4:**
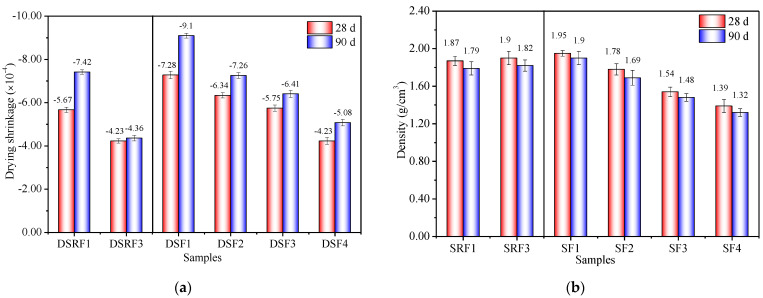
(**a**) Drying shrinkage and (**b**) density of alkali-activated samples with sisal leaf wastewater at 28 d and 90 d.

Figure 4 displays the drying shrinkage and density of the samples at 28 and 90 days. Incorporating WTSLW into the alkali activator resulted in a reduced drying shrinkage for the DSRF3 sample compared to DSRF1 at both 28 and 90 days. The drying shrinkage in DSRF3 also decreased with age, with a 41.24% lower drying shrinkage at 90 days compared to DSRF1. Similarly, using ATSLW as a complete replacement for the alkali activator led to an increased drying shrinkage over time but still resulted in a 44.18% lower drying shrinkage for DSF4 compared to DSF1 at 90 days. Drying shrinkage is determined by the dissolution–polymerization–hardening process of potentially active silicon–aluminum solid-waste materials in an alkaline environment [51,52,53]. Typically, alkali-activated waste materials exhibit higher drying shrinkage [54]. Soaking sisal leaves in water or alkali solutions leads to the dissolution of organic impurities due to biodegradation or erosion which, when mixed with the alkali-activated system, reduces the effective concentration of silicate–aluminate–sodium–calcium units per volume. The reduced probability of contact between particles and between particles and the solution limits the hydration or polymerization process, resulting in a lower drying shrinkage for samples with wastewater.

Additionally, the density of the samples decreased with age due to water loss during curing [8,54]. When 14% WTSLW replaced the alkali activator, the 90-day densities of the synthesized composites were close. However, when ATSLW fully replaced the activator, the 90-day density of the synthesized composites significantly decreased with increasing soaking duration. The 90-day density of SF4 was 30.53% lower than that of SF1. This decrease in density is attributed to the physical occupation of the liquid space in the alkali-activated system by a large number of organic substances from the sisal leaves. As the samples harden, these substances leave behind pores, resulting in a porous and lightweight composite material with the advantages of reduced shrinkage, increased porosity, and reduced weight [4,55]. The porous nature of these materials is further validated in Section 3.4. Furthermore, the 90-day density of SF4, recorded at 1.32 g/cm^3^, falls below the reference value of 1.45 g/cm^3^ for sisal-fiber-reinforced alkali-activated composites reported in prior studies [49]. The density of NaOH-activated sample SF1, which incorporates SP and FA, attains a level of 1.90 g/cm^3^ after 90 days. This value is found to be lower than the range of 2.00 to 2.30 g/cm^3^ observed in alkali-activated mortars that incorporate a blend of FA, soda residue, or carbide slag [36,54].

In summary, the substitution of alkali activators with WTSLW and ATSLW in the synthesis of alkali-activated composites leads to decreased drying shrinkage, enhanced porosity, and reduced weight, offering potential benefits in porous material performance.

### 3.3. Effects of Sisal Leaf Wastewater on Flexural and Compressive Strengths

**Figure 5:**
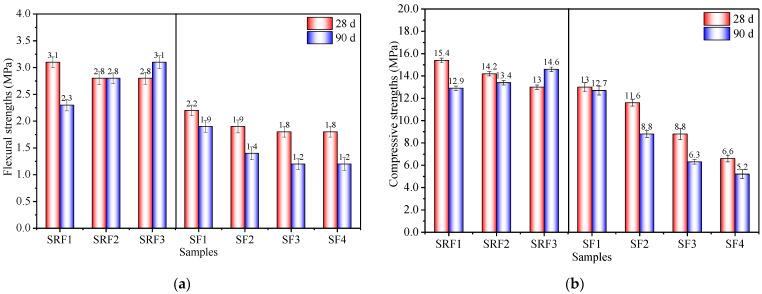
(**a**) Flexural strengths and (**b**) compressive strengths of alkali-activated samples with sisal leaf wastewater at 28 d and 90 d.

Figure 5 illustrates the flexural and compressive strengths of the samples at 28 and 90 days. Although the 28-day flexural and compressive strengths were lower with 14% WTSLW replacing the alkali activator, the 90-day strengths were higher. Specifically, the 90-day flexural and compressive strengths of SRF3 increased by 34.8% and 13.2%, respectively, compared to SRF1. The decrease in flexural and compressive strengths of alkali-activated SP-RM-FA composite materials over time is primarily due to rapid SP hydration, moisture loss during room temperature curing, and crack development [37,49]. However, the addition of 14% WTSLW, while causing slightly lower early strengths, continues to enhance long-term strengths, surpassing the control group. This is mainly because the soaking of sisal leaves in water leads to the dissolution of organic substances such as pectin, organic acids, sugars, and hemicellulose due to fermentation and corrosion [18]. WTSLW exhibits acidity (pH is 5.87, as described in Section 2.1). These organic substances undergo partial neutralization reactions with the alkaline environment (Na^+^, Ca^2+^, and OH^−^) to form stable substances like sodium organic salts or calcium organic salts. These substances provide Na^+^ and Ca^2+^ ions during the curing period, forming a gel structure with the alkali-activated product, which corresponds to the conclusion that alkali-activated materials can absorb harmful substances [56]. This indicates that 14% WTSLW as a substitute for the alkali activator in the synthesis of alkali-activated composites is beneficial for the development of flexural and compressive strengths in the later stages.

Conversely, when ATSLW replaces the alkali activator, the 90-day flexural and compressive strengths of SF4 decreased by 36.8% and 59.1%, respectively, compared to SF1. The main reason for this sharp decrease in strength with increasing soaking duration is the higher porosity and reduced bond strength between particles and gel in the material [57]. This is consistent with the conclusions of decreased density (Figure 4, Section 3.2) and increased porosity (Section 3.4) mentioned in this study. Previous work showed that substituting up to 25% of commercial NaOH with industrial alkaline wastewater from the Portuguese paper and pulp industry in the synthesis of AAMs does not negatively impact mechanical performance. It may maintain or even slightly enhance the compressive strength of the materials [58]. These variations in compressive strengths can be attributed to differences in the source of the wastewater and the chemical composition of the organic matter present. The organic content of sisal leaf wastewater is higher and more complex than the wastewater from the Portuguese paper and pulp industry.

In summary, WTSLW as a substitute for the alkali activator eliminates the strength regression phenomenon of composite materials over time, which is positively significant for the development of long-term strengths. Moreover, it provides new ideas for the development of new retarders. Although ATSLW reduces the flexural and compressive strengths of composite materials, it provides insights for the preparation of new foam composites using wastewater.

### 3.4. Effects of Sisal Leaf Wastewater on Morphology and Microstructure

**Figure 6:**
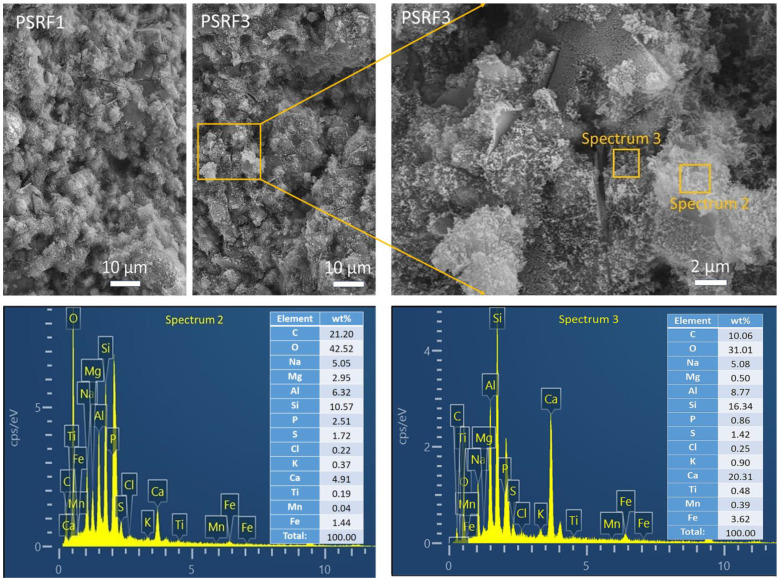
SEM images and EDS spectra of alkali-activated samples with WTSLW at 90 days.

The morphology and microstructure of alkali-activated composites containing 14% WTSLW were characterized using SEM and EDS analyses (Figure 6). The morphology of 90-day sample PSRF3, containing 14% WTSLW, was similar to that of the control sample PSRF1, which did not contain WTSLW. Both samples exhibited unreacted particles embedded in the structure, surrounded by gel-like material, which contributed to the strength of the composites. Upon further magnification of PSRF3, the gel-like material surrounding the particles was observed. The EDS analysis was performed on two micro-areas of the gel-like material, focusing on elements with a mass fraction greater than 2 wt%. Due to the heterogeneous nature of the composites, Mg and P elements were slightly higher than 2 wt% in Spectrum 2 and lower than 2 wt% in Spectrum 3 and are not discussed further in this article. The main elements detected in both Spectrum 2 and Spectrum 3 were O, C, Si, Al, Na, and Ca. The high content of O, Si, and Ca in Spectrum 3 suggested the formation of calcium silicate hydrate gel, while the addition of Al could lead to the formation of calcium aluminate hydrate [59,60]. In Spectrum 2, the significant increase in C and O compared to Spectrum 3 could be attributed to the carbonation reaction on the sample surface, forming carbonates, or the presence of organic salts from the organic impurities in 14% WTSLW reacting with the alkali solution in the hardened structure. Therefore, the incorporation of 14% WTSLW into the alkali-activated composites resulted in the formation of calcium silicate (aluminate) hydrate gel and did not have a negative impact on the microstructure of the materials.

**Figure 7:**
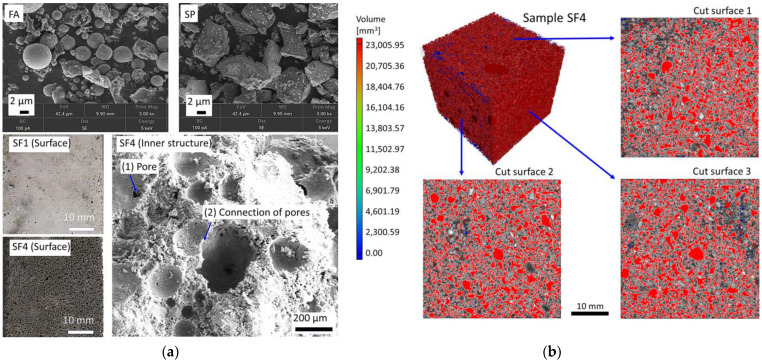
(**a**) SEM images and (**b**) CT micrograph of alkali-activated samples with ATSLW at 90 days.

Furthermore, Figure 7 characterizes the microstructure of alkali-activated composites synthesized using ATSLW as a replacement for the alkali activator. The granular binder materials FA and SP, under the activation of ATSLW, resulted in the formation of porous samples. As the soaking duration of sisal leaves increased, the surface porosity of the composites also increased significantly (Figure 7a). There were obvious open pores connecting at positions (1) and (2), and the CT image cross-section (Figure 7b) also showed the interconnection between pores. The introduction of ATSLW as an alkali activator replacement resulted in the largest proportion of macropores, with a total porosity of 43.6% for the SF4 composite. The pore-increasing effect of ATSLW was similar to that of vegetable oil [61]. With alkali activation, organic compounds and alkali solution were incorporated into the structure of the produced products, weakening the surface adhesion between particles and resulting in a reduction in bonding strengths (Figure 5, Section 3.3). After the hardening of alkali-activated composites, water is easily removed during the curing period, leaving a large network of interconnected pores [62].

In conclusion, both WTSLW and ATSLW, when used as replacements for the alkali activator in the synthesis of alkali-activated composites, resulted in the formation of calcium silicate (aluminate) hydrate gel, with ATSLW showing a specific effect of increasing interconnected porosity.

### 3.5. Effects of Sisal Leaf Wastewater on Mineral Compositions

**Figure 8:**
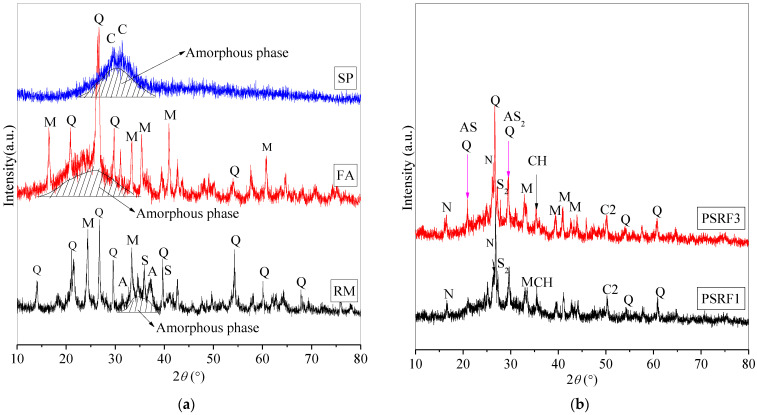
XRD patterns: (**a**) binder materials and (**b**) alkali-activated samples (PSRF1 and PSRF3).

The analysis of mechanical strength and microstructure in Figure 5 and Figure 6 indicated that substituting partial alkali activators with WTSLW could enhance the later-stage strength. To further understand the reaction mechanisms, a mineral composition analysis was conducted on samples PSRF1 and PSRF3, as shown in Figure 8 and Table 3.

Figure 8a illustrates that the binder SP is primarily composed of amorphous phases with some calcite crystals, which correspond to a broad non-diffractive hump peak between 22° and 40° 2θ. FA is mainly composed of quartz and mullite crystals, as well as a silica–aluminum-containing amorphous phase, corresponding to a hump peak range of 15° to 35° 2θ. The raw material RM consists mainly of crystalline phases such as quartz, mullite, calcium aluminate-hydrated (C-A-H), and calcium silicate-hydrated (C-S-H), along with some silica–aluminum amorphous phases, corresponding to hump peaks between 30° and 40° 2θ.

Figure 8b presents the mineral composition of samples PSRF1 and PSRF3 at 90 days. Compared to the binder materials, the mixture of SP, RM, and FA, when alkali-activated, formed new mineral phases including zeolite N-A-S-H, C-S-H, calcium hydroxide (Ca(OH)_2_), calcium carbonate (CaCO_3_), and two types of calcium silicate–aluminate hydrated (C-A-S-H, referred to as AS and AS_2_) in PSRF1. However, the C-S-H and C-A-H in the red mud (RM) disappeared, possibly due to the low extent of mixed reactions or because they were encapsulated by gel products.

When WTSLW was incorporated into the composite, no new mineral phases were formed in PSRF3 that were different from those in PSRF1, indicating that WTSLW did not affect the mineral composition of the alkali-activated composites. The formation of N-A-S-H occurred through the crystallization of an amorphous sodium–silica–aluminate polymer gel [63]. C-S-H and C-A-S-H were partly derived from the binder material RM and mainly from the hydration reaction products involving CaO, SiO_2_, Al_2_O_3_, and H_2_O [37]. Amorphous substances that were not detectable by XRD included amorphous sodium–silica–aluminate polymer gels, C-S-H gels, and C-A-S-H gels, which corresponded to hump peaks in the range of 22° to 36° 2θ. There was crystalline precipitation of Ca(OH)_2_ in an alkaline environment, but as the curing age increased, CO_2_ in the air reacted with Ca(OH)_2_ to form CaCO_3_ crystals [64], which is consistent with the elevated C and O content observed in the EDS measurements (Figure 6, Section 3.4).

In conclusion, at 90 days and excluding the effects of carbonation, the incorporation of 14% WTSLW into the SP-RM-FA-based alkali-activated material did not affect the mineral composition. The composite still consisted of amorphous N-A-S-H, C-S-H, and C-A-S-H gels coexisting with crystalline phases to produce cementation and form a solidified body.

### 3.6. Effects of Sisal Leaf Wastewater on Chemical Bonds and Gel Products

**Figure 9:**
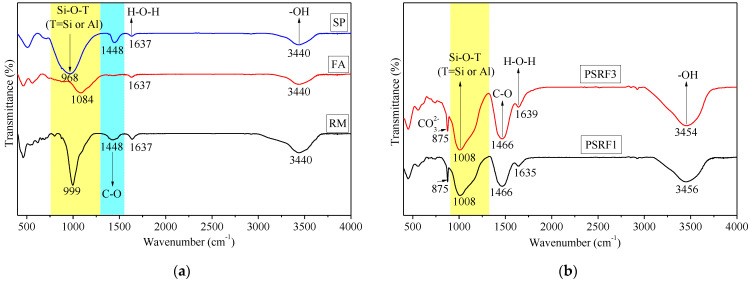
FTIR spectra: (**a**) binder materials, (**b**) alkali-activated samples (PSRF1 and PSRF3).

FTIR spectra in Figure 9 reveal the vibrational properties of chemical bonds in the sample PSRF1, sample PSRF3, and raw materials. A strong absorption peak at 875 cm^−1^ in PSRF1 and PSRF3, absent in raw materials, indicates new carbonate formation [65] following the alkali activation of SP, RM, FA, and NaOH. This peak corresponds to the bending vibration of the CO_3_^2−^ bond, suggesting the reaction of NaOH and Ca(OH)_2_ with atmospheric CO_2_ to form Na_2_CO_3_ and CaCO_3_, leading to carbonation.

The absorption peaks at 1448 cm^−1^ and 1466 cm^−1^ are attributed to the stretching vibration of the C-O bond [66], with the higher wavenumbers and increased intensities in PSRF1 and PSRF3 indicating increased carbonation. The absorption peak at 1008 cm^−1^ is associated with the asymmetric stretching vibration of the Si-O-T (Si or Al) bond in the silico-aluminate product [67,68] in Figure 9b. The shift in absorption peaks after alkali activation suggests the formation of Si-O-Si chains from amorphous Si-O and Al-O bonds in SP and RM, leading to C-S-H products [51]. In contrast, alkali activation drives FA towards the formation of Si-O-Al chains, resulting in silico-aluminate polymers [23]. The coexistence of silicate and silico-aluminate products in PSRF1 is consistent with the previous literature [69].

The absorption peaks at 1635, 1637, and 1639 cm^−1^ correspond to the bending vibration of the H-O-H bond in water molecules (Figure 9a,b), while the peaks at 3440 cm^−1^, 3446 cm^−1^, 3454 cm^−1^ (PSRF3), and 3455 cm^−1^ (PSRF1) are attributed to the stretching vibration of hydroxyl -OH groups [20,70]. No shift in peaks in PSRF3 indicates that the incorporation of WTSLW does not affect the binding of inorganic and organic components.

However, the main characteristic peaks of C-O bond stretching vibrations, aromatic ring vibration, and -CH_2_ vibration [71,72] related to cellulose, hemicellulose, and lignin do not appear in the FTIR spectra. The absence of these peaks suggests that the organic components leached from sisal leaves are effectively decomposed under the alkali activation process. At a pH level of 11 or higher, cell lysis takes place, which subsequently releases significant amounts of organic material into the alkaline solution [73].

At a 90-day curing age, excluding the carbonation, the addition of 14% WTSLW does not affect the composition and structure of the products of the SP-RM-FA-based alkali-activated material, as confirmed by XRD pattern analysis (Figure 8, Section 3.5).

### 3.7. Discussion

The present study focuses on the performance and mechanism of alkali-activated solid-waste-based composites synthesized by substituting NaOH activator solution with water- and alkali-treated wastewater from mature sisal leaves. It was observed that water-treated sisal leaf wastewater (WTSLW) partially replaces NaOH solution in alkali-activated materials and significantly enhances the flexural and compressive strengths at later stages. The 90-day flexural and compressive strengths of SRF3 (with 14% WTSLW) increased by 34.8% and 13.2%, respectively, compared to SRF1 (without WTSLW).

**Figure 10:**
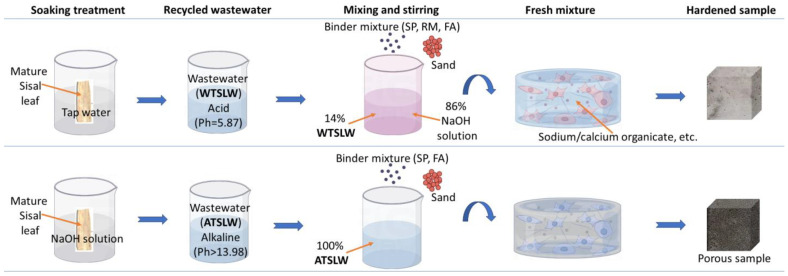
Schematic diagram of the recycling of WTSLW and ATSLW wastewater and the preparation of alkali-activated solid-waste-based samples.

A schematic diagram of synthesizing alkali-activated composites using sisal leaf wastewater is displayed in Figure 10. Organic acids, resulting from fermentation or decomposition and exhibiting acidity, are present in the organic impurities leached from mature sisal leaves soaked in water for five days [18]. The organic acids rapidly undergo neutralization chemical reactions upon encountering NaOH solution or CaO (containing Na^+^, Ca^2+^, and OH^−^) in the mixture, forming sodium or calcium organic salts. This not only reduces the alkalinity of the system but also decreases the rate of gelation and hardening of the fresh mixture, resulting in increased fluidity and lower early flexural and compressive strengths. However, as the alkali activation process continues, the organic salts, due to their soluble Na^+^ and Ca^2+^ cations, participate in the alkali activation process and become encapsulated in the silicate (aluminate) product gel [8,56]. This promotes a subsequent increase in the strength of the composites.

However, the role of alkali-treated sisal leaf wastewater (ATSLW) in preparing alkali-activated composites is significantly different from that of WTSLW. Due to the soaking of sisal leaves in a strongly alkaline environment (10 wt% NaOH for 8 ± 0.2 h), the recycled ATSLW exhibits strong alkalinity. ATSLW replaces NaOH solution to activate SP and FA, forming alkali-activated composites with certain strengths (90-day flexural strength of 1.2 MPa and compressive strength of 5.2 MPa). Additionally, the dissolved molecular organic substances (pectin, sugars, hemicellulose, etc.) weaken the surface-bonding characteristics between particles [74], leading not only to reduced flexural and compressive strengths but also to the formation of a large number of interconnected pores, compared to the controls (without ATSLW).

Previous studies [3] showed that a 15% NaOH solution is used to soak dead sisal for 24 h to obtain alkali-activated plant wastewater. The recycled plant wastewater is used to replace the alkali solution to activate a mixture of SP, RM, FA, and steel slag (with a mass ratio of 6:1:10:1). An improved two-step addition method is used for mixing, followed by mixing, stirring, molding, and curing, resulting in porous samples with a compressive strength of about 2.0 MPa. In this study, ATSLW is obtained by soaking mature sisal leaves in a 10 wt% NaOH solution for 8 ± 0.2 h, and ATSLW is used to replace the alkali solution to activate a mixture of SP and FA (with a mass ratio of 1:2). A one-step addition method is used for mixing, followed by mixing, stirring, molding, curing, and other processes, resulting in porous samples with a compressive strength of 5.2 MPa. Different treatment concentrations, different cementitious materials, and different mixing methods result in composite materials with different compressive strengths [75,76,77,78]. Therefore, there are many factors influencing the preparation of alkali-activated composites using ATSLW. However, it can be determined that ATSLW can always undergo alkali-activation gelation with potentially active silicon–aluminum raw materials (SP or FA, etc.), which is determined by the strong alkalinity of ATSLW. However, the recycling methods of WTSLW and ATSLW and their mixing methods with various solid wastes still lack standard references, which hinders the in-depth research process of synthesizing alkali-activated composites using plant wastewater, and this will be the direction of the next research.

## 4. Conclusions

This study involved the utilization of wastewaters derived from water and alkali treatment of mature sisal leaves as substitutes for alkali activators in the synthesis of alkali-activated solid-waste-based composites. Through the analysis of fresh properties, hardened properties, and microstructural characteristics, with a particular focus on the effects of the replacement amount of wastewater and the duration of alkali soaking, the role of mature sisal leaf wastewater was assessed. The primary conclusions are summarized as follows:

(1) The substitution of 14% water-treated sisal leaf wastewater (WTSLW) for the alkali activator (NaOH solution) in the synthesis of alkali-activated solid-waste-based composites, although delaying the early strength of the composite, was found to enhance the later-term flexural and compressive strengths and eliminate the strength regression with age.
(2) Mature sisal leaves were soaked in a 10 wt% NaOH solution for 8 ± 0.2 h to obtain alkali-treated sisal leaf wastewater (ATSLW). The use of ATSLW as a substitute for the alkali activator in the synthesis of alkali-activated solid-waste-based composites resulted in the production of porous composites (with a porosity of 43.6% and a compressive strength of 5.2 MPa after 90 days). This indicates that ATSLW possesses the capacity to activate binder materials and increase porosity.
(3) The substitution of both WTSLW and ATSLW for the alkali activator in the synthesis of alkali-activated solid-waste-based composites not only increases fluidity but also reduces the drying shrinkage. The DSRF3, with 14% WTSLW, exhibited a 41.24% reduction in drying shrinkage at 90 days compared to DSRF1 (without WTSLW). Similarly, DSF4, with ATSLW, resulted in a 44.18% reduction in drying shrinkage at 90 days compared to DSF1 (without ATSLW).
(4) At a 90-day curing age, excluding similar carbonation, the substitution of 14% WTSLW does not affect the composition and structure of the products of the SP-RM-FA-based alkali-activated material. The composite still consisted of amorphous N-A-S-H, C-S-H, and C-A-S-H gels coexisting with crystalline phases to produce cementation.

In summary, the recycling of both types of sisal leaf wastewater is beneficial in the alkali activation of various solid-waste-based composites. WTSLW improves the development of later-term strength, while ATSLW increases porosity and reduces drying shrinkage. Integrating wastewater, specifically WTSLW and ATSLW, into the synthesis of alkali-activated materials, offers a variety of benefits. These benefits include cost-effective wastewater treatment, improved mechanical properties of the composites, and significant environmental advantages. This approach presents a sustainable solution for managing industrial waste and contributes to the creation of valuable construction materials. As a result, it represents a promising area for further research and application. However, in the next phase of research, it is necessary to refine the levels of influencing factors and standardize the wastewater recycling methods to provide practical guidance for improving the performance of alkali-activated solid-waste-based composites.

## Figures and Tables

**Table 1:** Chemical components of binder materials by X-ray fluorescence method (mass%). ‘Non.’ notes no ingredients.

Components	Slag Powder (SP)	Red Mud (RM)	Fly Ash (FA)
SiO_2_	35.1	27.5	46.1
Al_2_O_3_	16.2	28.4	23.2
CaO	33.6	2.5	5.4
MgO	11.1	0.2	2.6
Fe_2_O_3_	*Non.*	25.8	8.0
K_2_O	*Non.*	0.1	*Non.*
SO_3_	*Non.*	0.8	0.8
Na_2_O	*Non.*	14.7	*Non.*
P_2_O_5_	*Non.*	*Non.*	0.6
TiO_2_	*Non.*	*Non.*	0.3
Others	4.1	*Non.*	1.5
Loss on ignition (1000 °C)	*Non.*	*Non.*	9.1

**Table 2:** Wastewater substitution and mixing proportion of alkali-activated composites based on slag powder (SP), red mud (RM), and fly ash (FA).

Sample No.	Binder Materials (g)	2 mol/L NaOH Solution (g)	WTSLW (g)—(mass%)	ATSLW (g)	Liquid– Binder Ratio	Binder– Sand Ratio	Soaking Duration (h)	Influencing Factors
SRF1	Mixture 1	570.0	0.0—(0%)	—	1:1.3	1:1.8	—	(WTSLW) Substitution
SRF2	Mixture 1	535.0	35.0—(7%)	—	1:1.3	1:1.8	—	
SRF3	Mixture 1	500.0	70.0—(14%)	—	1:1.3	1:1.8	—	
PSRF1	Mixture 1	540.0	0.0—(0%)	—	1:1.4	—	—	(WTSLW) Substitution
PSRF3	Mixture 1	464.4	75.6—(14%)	—	1:1.4	—	—	
DSRF1	Mixture 1	480.0	0.0—(0%)	—	1:1.6	1:2.0	—	(WTSLW) Substitution
DSRF3	Mixture 1	412.8	67.2—(14%)	—	1:1.6	1:2.0	—	
SF1	Mixture 2	—	—	172.5	1:4.3	1:3.0	0	(ATSLW) Soaking duration
SF2	Mixture 2	—	—	172.5	1:4.3	1:3.0	1 ± 0.2	
SF3	Mixture 2	—	—	172.5	1:4.3	1:3.0	5 ± 0.2	
SF4	Mixture 2	—	—	172.5	1:4.3	1:3.0	8 ± 0.2	
DSF1	Mixture 2	—	—	172.5	1:4.3	1:2.0	0.0	(ATSLW) Soaking duration
DSF2	Mixture 2	—	—	172.5	1:4.3	1:2.0	1 ± 0.2	
DSF3	Mixture 2	—	—	172.5	1:4.3	1:2.0	5 ± 0.2	
DSF4	Mixture 2	—	—	172.5	1:4.3	1:2.0	8 ± 0.2	

Note: Mixture 1 denotes the 750 g compound binders of 250 g SP, 50 g RM, and 450 g FA. Mixture 2 denotes the 750 g compound binders of 250 g SP and 500 g FA. WTSLW refers to water-treated sisal leaf wastewater. WTSLW mass percentage (mass%) refers to the mass ratio of WTSLW to NaOH solution. ATSLW refers to 10 wt% NaOH-treated sisal leaf wastewater.

**Table 3:** Mineral phases detected in Figure 8.

Abbreviations	Mineral Phases	pdf Card Number
Q	Quartz, SiO_2_	pdf# 97-003-9830
C	Calcite, CaCO_3_	pdf# 97-001-8166
M	Mullite, Si-Al-O	pdf# 00-029-1487
A	Calcium aluminate hydrated, C-A-H	pdf# 97-041-8966
S	Calcium silicate hydrated, C-S-H	pdf# 97-024-0406
S_2_	Calcium silicate hydrated, C-S-H	pdf# 97-034-0002
AS	Calcium aluminosilicate hydrated, C-A-S-H	pdf# 00-015-0179
AS_2_	Calcium aluminosilicate hydrated, C-A-S-H	pdf# 99-000-4228
C2	Calcium carbonate, CaCO_3_	pdf# 99-000-4172
N	Sodium aluminosilicate hydrated, N-A-S-H	pdf# 00-056-0499
CH	Calcium hydroxide, Ca(OH)_2_	pdf# 00-003-0865

## Data Availability

The original contributions presented in the study are included in the article, further inquiries can be directed to the corresponding author.
